# Individual differences in the learning potential of human beings

**DOI:** 10.1038/s41539-016-0003-0

**Published:** 2017-01-12

**Authors:** Elsbeth Stern

**Affiliations:** ETH Zürich, Clausiusstrasse 59, CH-8092 Zürich, Switzerland

## Abstract

To the best of our knowledge, the genetic foundations that guide human brain development have not changed fundamentally during the past 50,000 years. However, because of their cognitive potential, humans have changed the world tremendously in the past centuries. They have invented technical devices, institutions that regulate cooperation and competition, and symbol systems, such as script and mathematics, that serve as reasoning tools. The exceptional learning ability of humans allows newborns to adapt to the world they are born into; however, there are tremendous individual differences in learning ability among humans that become obvious in school at the latest. Cognitive psychology has developed models of memory and information processing that attempt to explain how humans learn (general perspective), while the variation among individuals (differential perspective) has been the focus of psychometric intelligence research. Although both lines of research have been proceeding independently, they increasingly converge, as both investigate the concepts of working memory and knowledge construction. This review begins with presenting state-of-the-art research on human information processing and its potential in academic learning. Then, a brief overview of the history of psychometric intelligence research is combined with presenting recent work on the role of intelligence in modern societies and on the nature-nurture debate. Finally, promising approaches to integrating the general and differential perspective will be discussed in the conclusion of this review.

## Human learning and information processing

In psychology textbooks, learning is commonly understood as the long-term change in mental representations and behavior as a result of experience.^1^ As shown by the four criteria, learning is more than just a temporary use of information or a singular adaption to a particular situation. Rather, learning is associated with changes in mental representations that can manifest themselves in behavioral changes. Mental and behavioral changes that result from learning must be differentiated from changes that originate from internal processes, such as maturation or illness. Learning rather occurs as an interaction with the environment and is initiated to adapt personal needs to the external world.

From an evolutionary perspective,^2^ living beings are born into a world in which they are continuously expected to accomplish tasks (e.g., getting food, avoiding threats, mating) to survive as individuals and as species. The brains of all types of living beings are equipped with instincts that facilitate coping with the demands of the environment to which their species has been adapted. However, because environments are variable, brains have to be flexible enough to optimize their adaptation by building new associations between various stimuli or between stimuli and responses. In the case of classical conditioning, one stimulus signals the occurrence of another stimulus and thereby allows for the anticipation of a positive or negative consequence. In the case of operant conditioning, behavior is modified by its consequence. Human beings constantly react and adapt to their environment by learning through conditioning, frequently unconsciously.^1^

However, there is more to human learning than conditioning, which to the best of our knowledge, makes us different from other species. All living beings must learn how to obtain access to food in their environment, but only human beings cook and have invented numerous ways to store and conserve their food. While many animals run faster than humans and are better climbers, the construction and use of vehicles or ladders is unique to humans. There is occasional evidence of tool use among non-human species passed on to the next generation,^3,4^ but this does not compare to the tools humans have developed that have helped them to change the world. The transition from using stonewedges for hunting to inventing wheels, cars, and iPhones within a time period of a few thousand years is a testament to the unique mental flexibility of human beings given that, to the best of our knowledge, the genes that guide human brain development have not undergone remarkable changes during the last 50,000 years.^5^ This means that as a species, humans are genetically adapted to accomplish requirements of the world as it existed at approximately 48,000 BC. What is so special about human information processing? Answers to this question are usually related to the unique resource of consciousness and symbolic reasoning abilities that are, first and foremost, practiced in language. Working from here, a remarkable number of insights on human cognition have been compiled in the past decades, which now allow for a more comprehensive view of human learning.

### Human learning from a general cognitive perspective

**Fig. 1:**
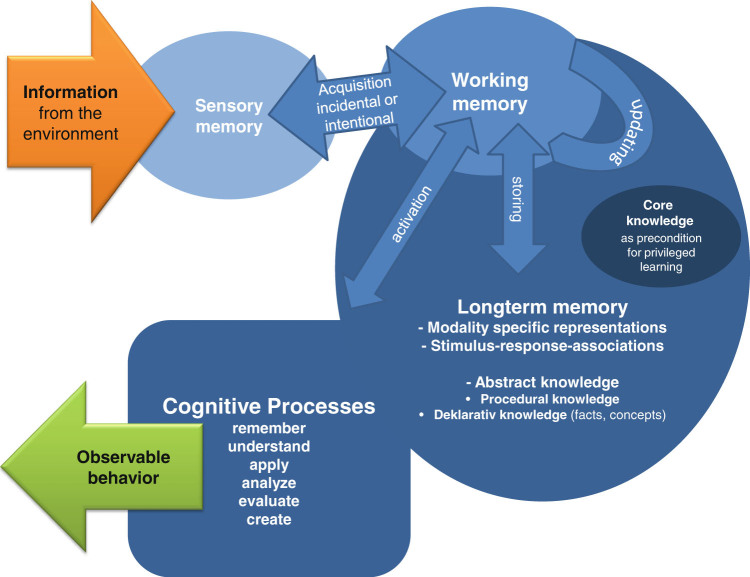
A model of human information processing, developed together with Dr. Lennart Schalk

Learning manifests itself in knowledge representations processed in memory. The encoding, storage, and retrieval of information have been modeled in the multi-store model of human memory depicted in Fig. 1.^6^ Sensory memory is the earliest stage of processing the large amount of continuously incoming information from sight, hearing, and other senses. To allow goal-directed behavior and selective attention, only a fractional amount of this information passes into the working memory, which is responsible for temporarily maintaining and manipulating information during cognitive activity.^7,8^ Working memory allows for the control of attention and thereby enables goal-directed and conscious information processing. It is the gatekeeper to long-term memory, which is assumed to have an unlimited capacity. Here, information acquired through experience and learning can be stored in different modalities as well as in symbol systems (e.g., language, script, mathematical notation systems, pictorials, music prints).

The multi-store model of human information processing is not a one-way street, and long-term memory is not to be considered a storage room or a hard-disk where information remains unaltered once it has been deposited. A more appropriate model of long-term memory is a self-organizing network, in which verbal terms, images, or procedures are represented as interlinked nodes with varying associative strength.^9^ Working memory regulates the interaction between incoming information from sensory memory and knowledge activated from long-term memory. Very strong incoming stimuli (e.g., a loud noise or a harsh light), which may signal danger, can interrupt working memory activities. For the most part, however, working memory filters out irrelevant and distracting information to ensure that the necessary goals will be achieved undisturbed. This means that working memory is continuously selecting incoming information, aligning it with knowledge retrieved from long-term memory, and preparing responses to accomplishing requirements demanded by the environment or self-set goals. Inappropriate and unsuitable information intruding from sensory as well as from long-term memory has to be inhibited, while appropriate and suitable information from both sources has to be updated.^8^ The strength with which a person pursues a particular goal has an impact on the degree of inhibitory control. In case of intentional learning, working memory guards more against irrelevant information than in the case of mind wandering. Less inhibitory control makes unplanned and unintended learning possible (i.e., incidental learning).

These working memory activities are permanently changing the knowledge represented in long-term memory by adding new nodes and by altering the associative strength between them. The different formats knowledge can be represented in are listed in Fig. 1; some of them are more closely related to sensory input and others to abstract symbolic representations. In cognitive psychology, learning is associated with modifications of knowledge representations that allow for better use of available working memory resources. Procedural knowledge (knowing how) enables actions and is based on a production-rule system. As a consequence of repeated practice, the associations between these production rules are strengthened and will eventually result in a coordinated series of actions that can activate each other automatically with a minimum or no amount of working memory resources. This learning process not only allows for carrying out the tasks that the procedural knowledge is tailored to perform more efficiently, but also frees working memory resources that can be used for processing additional information in parallel.^10–12^

Meaningful learning requires the construction of declarative knowledge (knowing that), which is represented in symbol systems (language, script, mathematical, or visual-spatial representations). Learning leads to the regrouping of declarative knowledge, for instance by chunking multiple unrelated pieces of knowledge into a few meaningful units. Reproducing the orally presented number series “91119893101990” is beyond working memory capacity, unless one detects two important dates of German history: the day of the fall of the Berlin Wall: 9 November 1989 and the day of reunification: 3 October 1990. Individuals who have stored both dates and can retrieve them from long-term memory are able to chunk 14 single units into two units, thereby freeing working memory resources. Memory artists, who can reproduce dozens of orally presented numbers have built a very complex knowledge base that allows for the chunking of incoming information.^13^

Learning also manifests itself in the extension of declarative knowledge using concept formation and inferential reasoning. Connecting the three concepts of “animal, produce, milk” forms a basic concept of cow. Often, concepts are hierarchically related with superordinate (e.g., animal) and subordinate (e.g., cow, wombat) ordering. This provides the basis for creating meaningful knowledge by deductive reasoning. If the only thing a person knows about a wombat is that it is an animal, she can nonetheless infer that it needs food and oxygen. Depending on individual learning histories, conceptual representations can contain great variations. A farmer’s or a veterinarian’s concept of a cow is connected to many more concepts than “animal, produce, milk” and is integrated into a broader network of animals. In most farmers’ long-term memory, “cow” might be strongly connected to “pig”, while veterinarians should have particularly strong links to other ruminants. A person’s conceptual network decisively determines the selection and representation of incoming information, and it determines the profile of expertise. For many academic fields, first and foremost in the STEM area (Science, Technology, Engineering, Mathematics), it has been demonstrated that experts and novices who use the same words may have entirely different representations of their meaning. This has been convincingly demonstrated for physics and particularly in the area of mechanics.^14^ Children can be considered universal novices;^15^ therefore, their everyday concepts are predominantly based on characteristic features while educated adults usually consider defining features,^16–18^ as the example of “island” demonstrates. For younger children, it primarily refers to a warm place where one can spend ones’ holidays. In contrast, adults’ concept of island does refer to a tract of land that is completely surrounded by water but not large enough to be considered a continent.

The shift from characteristic to defining features is termed “conceptual change”,^16^ and promoting this kind of learning is a major challenge for school education. Students’ understanding of central concepts in an academic subject can undergo fundamental changes (e.g., the concept of weight in physics). Younger elementary school children often agree that a pile of rice has weight, but they may also deny that an individual grain of rice has weight at all. This apparently implausible answer is understandable given that younger children consider the concepts of “weight” and “being heavy” as equivalent. As such, children tend to agree that a grain of rice has weight if it is put on an ant’s back.^16^ As a consequence of their education, students usually understand that an object’s weight is determined with the assistance of scales and not necessarily by personal sensation. However, representing weight as the property of an object is still not compatible with scientific physics in the Newtonian sense by which weight is conceptualized as a relation between objects. Understanding weight in this sense requires an interrelated network of knowledge, including the concepts of force, gravity, and mass (among others).

As a result of classroom instruction, students are expected to acquire procedural and conceptual knowledge of the subjects they were taught. While procedures emerge as a function of repetition and practice, the acquisition of advanced concepts, which are consistent with state of the art science, is less straightforward.^14,19^ To support this kind of conceptual learning, insights from cognitive learning research have been integrated into educational research and are increasingly informing classroom practice. Several instructional methods have been developed and evaluated that support students in restructuring and refining their knowledge and thereby promote appropriate conceptual understanding, including self-explanations,^20^ contrasting cases,^21,22^ and metacognitive questions.^23^ Cognitive research has also informed the development of the “taxonomy of learning objects”.^24^ This instrument is widely employed for curriculum development and in teacher training programs to support the alignment of content-specific learning goals, means of classroom practice, and assessment. The taxonomy acknowledges the distinction between procedural and conceptual knowledge and includes six cognitive processes (listed in Fig. 1) that describe how knowledge can be transformed into observable achievement.

### How core knowledge innate to humans can meet with academic learning

What makes humans efficient learners, however, goes beyond general memory functions discussed so far. Similar to other living beings, humans do not enter the world as empty slates^2^ but are equipped with so-called core knowledge (Fig. 1). Evidence for core knowledge comes from preferential looking experiments with infants who are first habituated to a particular stimulus or scenario. Then, the infant is shown a second scenario that differs from the first in a specific manner. If the time he or she looks at this stimulus exceeds the looking-time at the end of the habituation phase of the first stimulus, this suggests that the infant can discriminate between the stimuli. This paradigm helps to determine whether infants detect violations of principles that underlie the physical world, such as the solidity of objects, where an object cannot occupy the same space as another object.^25,26^ Core knowledge, which allows privileged learning and behavioral functioning with little effort, also guides the unique human ability of symbolic communication and reasoning, first and foremost, langue learning.^27,28^ It is uncontested that humans are born with capacities for language learning, which includes the awareness of phonological, grammatical, and social aspects of language.^4,29,30^

Core knowledge can serve as a starting point for the acquisition of content knowledge that has emerged as a result of cultural development. This has been examined in detail for numerical and mathematical reasoning. Two core systems have been detected in infants. As early as at 6 months of age, infants show an ability for the approximate representations of numerical magnitude, which allow them to discriminate two magnitudes depending on their ratio.^31^ At the same age, the system of precise representations of distinct individuals allows infants to keep track of changes in small sets of up to three elements.^32^ Mathematical competencies emerge as a result of combining both core systems and linking them to number words provided by the respective culture.^33^ The Arabic place value number system, which is now common in most parts of the world, was only developed a few 100 years ago. Only after the number “0” had made its way from India via the Arabic countries to Europe were the preconditions for developing our decimal system available.^34^ The Arabic number system opened up the pathway to academic mathematics. Cultural transformations based on invented symbol systems were the key to advanced mathematics. Today’s children are expected to understand concepts within a few years of schooling that took mankind centennials to develop. Central content areas in mathematics curricula of high schools, such as calculus, were only developed less than three centuries ago.^35^ Given the differences between the Arabic and the Roman number systems, children born 2000 years ago could not make use of their numerical core knowledge in the same way today’s children can.

Core knowledge about navigation is meant to guide the acquisition of geometry, an area involved in numerous academic fields.^36,37^ The cornerstone of cultural development was the invention of writing, in which language is expressed by letters or other marks. Script is a rather recent cultural invention, going back approximately 5,000 years, whereas the human genome emerged approximately 50,000 years ago.^38^ Clearly, unlike oral language, humans are not directly prepared for writing and reading. Nonetheless, today, most 6-year-old children become literate during their 1st years of schooling without experiencing major obstacles. Human beings are endowed with the many skills that contribute to the ability to write and read, such as, first and foremost, language as well as auditory and visual perception and drawing. These initially independent working resources were coopted when script was invented, and teaching children to write and read at school predominantly means supporting the development of associations among these resources.^39^

Part of the core knowledge innate to humans has also been found in animals, for instance numerical knowledge and geometry, but to the best of our knowledge, no other animals have invented mathematics.^40^ Only humans have been able to use core knowledge for developing higher order cognition, which serves as a precondition for culture, technology, and civilization. Additionally, the unique function of human working memory is the precondition for the integration of initially independent representational systems. However, the full potential of working memory is not in place at birth, but rather matures during childhood and undergoes changes until puberty.^41^ Children under the age of two are unable to switch goals^42^ and memorize symbol representations appropriately.^43^

To summarize what has been discussed so far, there are two sources for the exceptional learning capacity of humans. The first is the function of working memory as a general-purpose resource that allows for holding several mental representations simultaneously for further manipulation. The second is the ancient corpus of the modularized core knowledge of space, quantities, and the physical and social world. Working memory allows for the connection of this knowledge to language, numerals, and other symbol systems, which provides the basis for reasoning and the acquisition of knowledge in academic domains, if appropriate learning opportunities are provided. Both resources are innate to human beings, but they are also sources of individual differences, as will be discussed in the following sections.

## Learning potentials are not alike among humans: the differential perspective

In the early twentieth century, a pragmatic need for predicting the learning potential of individuals initiated the development of standardized tests. The Frenchman Alfred Binet, who held a degree in law, constructed problems designed to determine whether children who did not meet certain school requirements suffered from mental retardation or from behavioral disturbances.^44^ He asked questions that still resemble items in today’s intelligence tests; children had to repeat simple sentences and series of digits forwards and backwards as well as define words such as “house” or “money”. They were asked in what respect a fly, an ant, a butterfly and a flea are alike, and they had to reproduce drawings from memory. William Stern, an early professor of psychology at the newly founded University of Hamburg/Germany, intended to quantify individual differences in intelligence during childhood and adolescence by developing the first formula for the intelligence quotient (IQ):^45^ IQ = Mental age/chronological age*100. Mental age refers to the average test score for a particular age group; this means that a 6-year-old child would have an IQ = 133 if their test score was equivalent to the mean score achieved in the group of 8-year-olds. From adolescence on, however, the average mental age scores increasingly converge, and because of the linear increase in chronological age, the IQ would decline—a trend that obviously does not match reality.

Psychologists from the United States, specifically headed by the Harvard and later Yale professor Robert Yerkes, decided to look at a person’s score relative to other people of the same age group. The average test score was assigned to an IQ = 100 by convention, and an individual’s actual score is compared to this value in terms of a standard deviation, an approach that has been retained to this day. World War I pushed the development of non-verbal intelligence tests, which were used to select young male immigrants with poor English language skills for military service.^46^ In the UK, the educational psychologist Cyril Burt promoted the use of intelligence tests for assigning students to the higher academic school tracks.^47^ Charles Spearman from the University College London was among the first to focus on the correlations between test items based on verbal, numerical, or visual-spatial content.^48^ The substantial correlations he found provided evidence for a general intelligence model (factor-g), which has been confirmed in the following decades by numerous studies performed throughout the world.^49^

The high psychometric quality of the intelligence tests constructed in different parts of the world by scientists in the early decades of the twentieth century have influenced research ever since. In 1923, Edward Boring, a leading experimental psychologist concluded, “Intelligence is what the tests test. This is a narrow definition, but it is the only point of departure for a rigorous discussion of the tests. It would be better if the psychologists could have used some other and more technical term, since the ordinary connotation of intelligence is much broader. The damage is done, however, and no harm need result if we but remember that measurable intelligence is simply what the tests of intelligence test, until further scientific observation allows us to extend the definition.”(ref. 50, p. 37). More than 70 years later, psychologists widely agreed on a definition for intelligence originally offered by Linda Gottfredsonin 1997: “Intelligence is a very general mental capability that, among other things, involves the ability to reason, plan, solve problems, think abstractly, comprehend complex ideas, learn quickly, and learn from experience. It is not merely book learning, a narrow academic skill, or test-taking smarts. Rather, it reflects a broader and deeper capability for comprehending our surroundings—‘catching on,’ ‘making sense’ of things, or ‘figuring out’ what to do” (ref. 51, p. 13). This definition is in line with the substantial correlations between intelligence test scores and academic success,^52^ whereas correlations with measures of outside-school success, such as income or professional status, are lower but still significant.^53,54^ Numerous longitudinal studies have revealed that IQ is a fairly stable measure across the lifespan, which has been most convincingly demonstrated in the Lothian Birth Cohorts run in Scotland. Two groups of people born in 1921 and 1936 took a test of mental ability at school when they were 11 years old. The correlation with IQ tests taken more than 60 years later was highly significant and approached *r* = .70 (ref. 55). The same data set also demonstrated a substantial long-term impact of intelligence on various factors of life success, among them career aspects, health, and longevity.^56^

Intelligence tests scores have proven to be objective, reliable, and valid measures for predicting learning outcome and more general life success. At the same time, the numerous data sets on intelligence tests that were created all over the world also contributed to a better understanding of the underlying structure of cognitive abilities. Although a factor g could be extracted in almost all data sets, correlations between subtests varied considerably, suggesting individual differences beyond general cognitive capabilities. Modality factors (verbal, numerical, or visual spatial) have been observed, showing increased correlations between tests based on the same modality, but requiring different mental operations. On the other hand, increased correlations were also observed between tests based on different modalities, but similar mental operations (e.g., either memorizing or reasoning). The hierarchical structure of intelligence, with factor g on the top and specific factors beneath, was quite obvious from the very beginning of running statistical analyses with intelligence items. Nonetheless, it appeared a major challenge for intelligence researchers to agree on a taxonomy of abilities on the second and subsequent levels. In 1993, John Carroll published his synthesis of hundreds of published data sets on the structure of intelligence after decades of research.^57^ In his suggested three-stratum model, factor g is the top layer, with the middle layer encompassing broader abilities such as comprehension knowledge, reasoning, quantitative knowledge, reading and writing, and visual and auditory processing. Eighty narrower abilities, such as spatial scanning, oral production fluency, and sound discrimination, are located in the bottom layer. To date, Carroll’s work is considered the most comprehensive view of the structure of individual variations in cognitive abilities.^58^ However, the interpretation of factor g is still under discussion among scientists. Factor g could be a comprehensive characteristic of the brain that makes information processing generally more or less efficient (top-down-approach). Existing data sets, however, are also compatible with a model of intelligence according to which the human brain is comprised of a large number of single abilities that have to be sampled for mental work (bottom-up approach). In this case, factor g can be considered a statistical correlate that is an emerging synergy of narrow abilities.^59^

### Genetic sources of individual differences in intelligence

From studies with identical and fraternal twins, we know that genetic differences can explain a considerable amount of variance in IQ. The correlation between test scores of identical twins raised together approaches *r* = .80 and thereby is almost equal to the reliability coefficient of the respective test. On the other hand, IQ-correlations between raised-together same-sex fraternal twins are rarely higher than .50, a value also found for regular siblings. Given that the shared environment for regular siblings is lower than for fraternal twins, this result qualifies the impact of environmental factors on intelligence. The amount of genetic variance is judged in statistical analyses based on the difference between the intra-pair correlations for identical and fraternal twins.^60^ High rates of heritability, however, do not mean that we can gauge a person’s cognitive capabilities from his or her DNA. The search for the genes responsible for the expression of cognitive capabilities has not yet had much success, despite the money and effort invested in human genome projects. It is entirely plausible that intelligence is formed by a very large number of genes, each with a small effect, spread out across the entire genome. Moreover, these genes seem to interact in very complicated ways with each other as well as with environmental cues.^61^

An entirely false but nonetheless still widespread misunderstanding is to equate “genetic sources” with “inevitability” because people fail to recognize the existence of reaction norms, a concept invented in 1909 by the German biologist, Richard Woltereck. Reaction norms depict the range of phenotypes a genotype can produce depending on the environment.^62^ For some few physiological individual characteristics (e.g., the color of eyes) the reaction norm is quite narrow, which means gene expression will rarely be affected by varying environments. Other physiological characteristics, such as height, have a high degree of heritability and a large reaction norm. Whether an individual reaches the height made possible by the genome depends on the nutrition during childhood and adolescence. In a wealthy country with uniform access to food, average height will be larger than in a poor country with many malnourished inhabitants. However, within both countries, people vary in height. The heritability in the wealthy country can be expected to approach 100% because everybody enjoyed sufficient nutrition. In contrast, in the poor country, some were sufficiently nourished and, therefore, reached the height expressed by their genome, while others were malnourished and, therefore, remained smaller than their genes would have allowed under more favorable conditions. For height, the reaction norm is quite large because gene expression depends on nutrition during childhood and adolescence. This explains the well-documented tendency for people who have grown up in developed countries to become progressively taller in the past decades.

The environment regulates gene expression, which means that instead of “nature vs. nurture”, a more accurate phrase is “nature via nurture”.^63^ The complex interaction between genes and environment can also explain the fact that heritability of intelligence increases during the lifespan.^61^ This well-established finding is a result of societies in which a broad variety of cognitive activities available in professional and private life enable adults more than children to actively select special environments that fit their genes. People who have found their niche can perfect their competencies by deliberate learning.

In the first decades of developing intelligence tests, researchers were naive to the validity of non-verbal intelligence; so-called culture-free or culture-fair tests, based on visual-spatial material such as mirror images, mazes or series and matrices of geometric figures, were supposed to be suitable for studying people of different social and cultural levels.^64^ This is now considered incorrect because in the meantime, there has been overwhelming evidence for the impact of schooling on the development of intelligence and the establishment and stabilization of individual differences. Approximately 10 years of institutionalized education is necessary for the intelligence of individuals to approach its maximum potential.^65–67^

Altogether, twin and adoption studies suggest that 50–80% of IQ variation is due to genetic differences.^61^ This relatively large range in the percentage across different studies is due to the heritability of intelligence in the population studied, specifically, the large reaction norm of the genes giving rise to the development of intelligence. Generally, the amount of variance in intelligence test scores explained by genes is higher the more society members have access to school education, health care, and sufficient nutrition. There is strong evidence for a decrease in the heritability of intelligence for children from families with lower socioeconomic status (SES). For example, lower SES fraternal twins resembled each other more than higher SES ones, indicating a stronger impact of shared environment under the former condition.^68^ In other words, because of the less stimulating environment in lower SES families, the expression of genes involved in the development of intelligence is likely to be hampered. Although it may be counterintuitive at first, this suggests that a high heritability rate of intelligence in a society is an indicator of economic and educational equity. Additionally, this means that countries that ensure access to nutrition, health care, and high quality education independent of social background enable their members to develop their intelligence according to their genetic potential. This was confirmed by a meta-analysis on interactions between SES and heritability rate. While studies run in the United States showed a positive correlation between SES and heritability rate, studies from Western Europe countries and Australia with a higher degree of economic and social equality did not.^69,70^

## Cognitive processes behind intelligence test scores: how individuals differ in information processing

In the first part of this paper, cognitive processes were discussed that, in principle, enable human beings to develop the academic competencies that are particularly advantageous in our world today. In the second part, intelligence test scores were shown to be valid indicators of academic and professional success, and differences in IQ were shown to have sound genetic sources. Over many decades, research on cognitive processes and psychometric intelligence has been developing largely independently of one another, but in the meantime, they have converged. Tests that were developed to provide evidence for the different components of human cognition revealed large individual differences and were substantially correlated with intelligence tests. Tests of memory function were correlated with tests of factor g. Sensory memory tests have shown that the exposure duration required for reliably identifying a simple stimulus (inspection time) is negatively correlatedwith intelligence.^71^ For working memory, there is a large body of research indicating substantial relationships between all types of working memory functions and IQ, with average correlations >.50 (refs 72–74). In these studies, working memory functions are measured by speed tasks that require goal-oriented active monitoring of incoming information or reactions under interfering and distracting conditions. Neural efficiency has been identified as a major neural characteristic of intelligence; more intelligent individuals show less brain activation (measured by electroencephalogram or functional magnetic resonance imaging) when completing intelligence test items ^75,76^ as well as working memory items.^77^ Differences in information-processing efficiency were already found in 4-month-old children. Most importantly, they could predict psychometric intelligence in 8-year-old children.^78^

These results clearly suggest that a portion of individual differences can be traced back to differences in domain-general cognitive competencies. However, psychometric research also shows that individual differences do exist beyond factor g on a more specific level. Differences in numerical, language, and spatial abilities are well established. Longitudinal studies starting in infancy suggest that sources of these differences may be traced back to variations in core knowledge. Non-symbolic numerical competencies in infancy have an impact on mathematical achievement.^79^ Similar long-term effects were found for other areas of core knowledge,^80^ particularly language.^81^

Endowed with general and specific cognitive resources, human beings growing up in modern societies are exposed to informal and formal learning environments that foster the acquisition of procedural as well as declarative knowledge in areas that are part of the school curriculum. Being endowed with genes that support efficient working memory functions and that provide the basis for usable core knowledge allows for the exploitation of learning opportunities provided by the environment. This facilitates the acquisition of knowledge that is broad as well as deep enough to be prepared for mastering the, as of yet, unknown demands of the future.^18^ Regression analyses based on longitudinal studies have revealed that the confounded variance of prior knowledge and intelligence predicts learning outcome and expertise better than each single variable.^82–84^ Importantly, no matter how intelligent a person is, gaining expertise in a complex and sophisticated field requires deliberate practice and an immense investment of time.^85^ However, intelligence differences will come into play in the amount of time that has to be invested to reach a certain degree of expertise.^86^ Moreover, intelligence builds a barrier to content areas in which a person can excel. As discussed in the first part of this paper, some content areas—first and foremost from STEM fields—are characterized by abstract concepts mainly based on defining features, which are themselves integrated into a broader network of other abstract concepts and procedures. Only individuals who clearly score above average on intelligence tests can excel in these areas.^84,87^ For individuals who were fortunate enough to attend schools that offered high-quality education, intelligence and measures of deep and broad knowledge are highly correlated.^88,89^ A strong impact of general intelligence has also been shown for university entrance tests such as the SAT, which mainly ask for the application of knowledge in new fields.^90,91^ Societies that provide uniform access to cognitively stimulating environments help individuals to achieve their potential but also bring to bear differences in intelligence. Education is not the great equalizer, but rather generates individual differences rooted in genes.
